# A High Performance Delta-Sigma Modulator for Neurosensing

**DOI:** 10.3390/s150819466

**Published:** 2015-08-07

**Authors:** Jian Xu, Menglian Zhao, Xiaobo Wu, Md. Kafiul Islam, Zhi Yang

**Affiliations:** 1 Department of Electrical and Computer Engineering, National University of Singapore, 4 Engineering Drive 3, Singapore 117576, Singapore; E-Mails: kafiul_islam@nus.edu.sg (M.K.I.); eleyangz@nus.edu.sg (Z.Y.); 2 Department of Biomedical Engineering, University of Minnesota Twin Cities, Minneapolis, MN 55455, USA; 3 Institute of VLSI Design, Zhejiang University, 38 Zheda Road, Xihu District, Hangzhou 310027, China; E-Mails: zhaoml@vlsi.zju.edu.cn (M.Z.); wuxb@vlsi.zju.edu.cn (X.W.)

**Keywords:** sensor interface, dynamic range, multi-bit quantizer, switched op-amp, Delta-Sigma modulator

## Abstract

Recorded neural data are frequently corrupted by large amplitude artifacts that are triggered by a variety of sources, such as subject movements, organ motions, electromagnetic interferences and discharges at the electrode surface. To prevent the system from saturating and the electronics from malfunctioning due to these large artifacts, a wide dynamic range for data acquisition is demanded, which is quite challenging to achieve and would require excessive circuit area and power for implementation. In this paper, we present a high performance Delta-Sigma modulator along with several design techniques and enabling blocks to reduce circuit area and power. The modulator was fabricated in a 0.18-μm CMOS process. Powered by a 1.0-V supply, the chip can achieve an 85-dB peak signal-to-noise-and-distortion ratio (*SNDR*) and an 87-dB dynamic range when integrated over a 10-kHz bandwidth. The total power consumption of the modulator is 13 μW, which corresponds to a figure-of-merit (*FOM*) of 45 fJ/conversion step. These competitive circuit specifications make this design a good candidate for building high precision neurosensors.

## 1. Introduction

**Figure 1:**
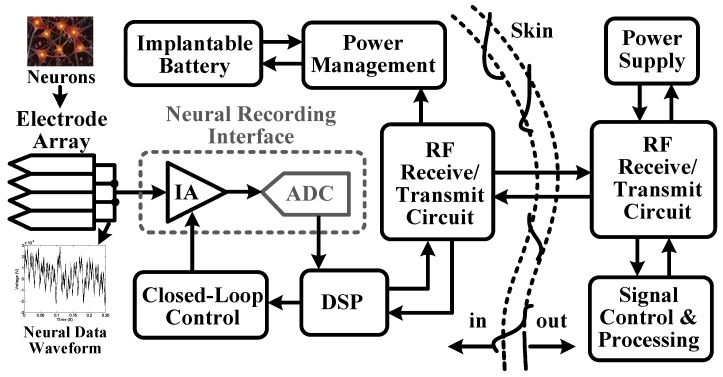
Block diagram of a wireless neural recording system.

Growing concerns for human health have stimulated the development of biomedical devices [1,2,3,4,5,6,7,8,9,10,11,12,13,14,15,16,17], such as biosensors for recording neural spikes, field potentials, electroencephalography (EEG), electrocardiography (ECG), electromyography (EMG), and so on. Figure 1 shows the block diagram in a wireless neural recording microsystem [1], where an analog-to-digital converter (ADC) is used for digitizing neural data recorded at each electrode. These neural data include several components, like local field potentials (LFPs), extracellular spikes and motion artifacts [18,19,20,21,22]. To record these neural data without saturating from large artifacts, it requires a wide dynamic range for data acquisition. The requirement is further pushed by the need to support more sophisticated neuroscience experiments and clinical applications, where motion artifacts tend to be more frequent and severe [23]. In this regard, it is important to integrate a high precision ADC in the recording microsystem.

In this paper, we present a Delta-Sigma ADC for building high precision recording microsystems. Compared to recent Delta-Sigma ADC designs [24,25,26,27,28,29], several techniques are used to improve circuit performance in terms of power, precision, area and *FOM*. (1) We have used high density metal-oxide-semiconductor (MOS) capacitors to replace metal-insulator-metal (MIM) capacitors for reducing circuit area [30]. As detailed in Section 4 and Section 5, circuit bias and integrator output swing are carefully designed, such that the distortion caused by MOS capacitors can be negligible. (2) We have adopted a multi-bit switched op-amp (MBSO)-based modulator architecture. Compared to those non-op-amp-based designs reported previously [25], our design offers a competitive performance in terms of power and area, maintaining less design complexity and restriction on precision [31].

To implement the proposed MBSO-based architecture, several circuit techniques have been incorporated as summarized below. First, we have performed careful characterization with each type of MOS capacitor. Based on the simulation results, we have chosen the optimal type of capacitor for each integrator. Second, a switched op-amp technique has been used where amplifiers are automatically turned off when they are not in use. Realizing such high performance switched op-amp depends on the bias circuits. In this design, novel bias circuits have been proposed to reduce switching power loss. Third, the static power of the quantizer has been removed through an elegant circuit arrangement. Finally, a new half-cycle operating resonator scheme has been designed for reducing in-band quantization noise. These techniques together boost the performance of the modulator and make it a competitive candidate for building high precision recording microsystems.

The rest of this paper is organized as follows. Section 2 analyzes the dynamic range of neural data using different sequences recorded from *in vivo* preparations and epilepsy patients. Section 3 gives a brief overview of different ADC structures. Section 4 presents the design analysis for the proposed Delta-Sigma modulator. The circuit implementation is shown in Section 5, followed by measurement results in Section 6. Section 7 gives the concluding remarks of this paper.

## 2. *In Vivo* Neural Data Dynamic Range Analysis

**Figure 2:**
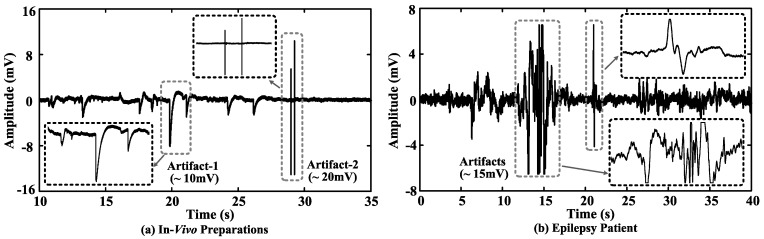
(**a**) *In vivo* neural recording from a rat preparation and (**b**) an intracortical recording from an epilepsy patient.

*In vivo* neural data recorded from extracellular space consist of both LFPs and extracellular spikes. The amplitude of extracellular spikes is inversely proportional to the distance between the recording site and neuron [32]. Neural spikes are from a few μV (the recording site is about 300 μm away from the neuron) to several hundred μV (the recording site is closest to the neuron), and LFPs are from tens of μV to several mV [32]. However, as shown in Figure 2, motion artifacts can be much larger, which would cause misinterpretation of the signals and sometimes saturate the recording electronics.

To study the required system dynamic range to accommodate both neural activities and artifacts, we have analyzed data sequences obtained from different setups: (1) *in vivo* preparations sampled at 40 kHz; (2) full-spectrum recordings from epilepsy patients sampled at 32 kHz. Table 1 summarizes the averaged data dynamic range with/without artifacts from different sequences, where the artifacts and spikes are manually labeled by a domain expert.

**Figure 3:**
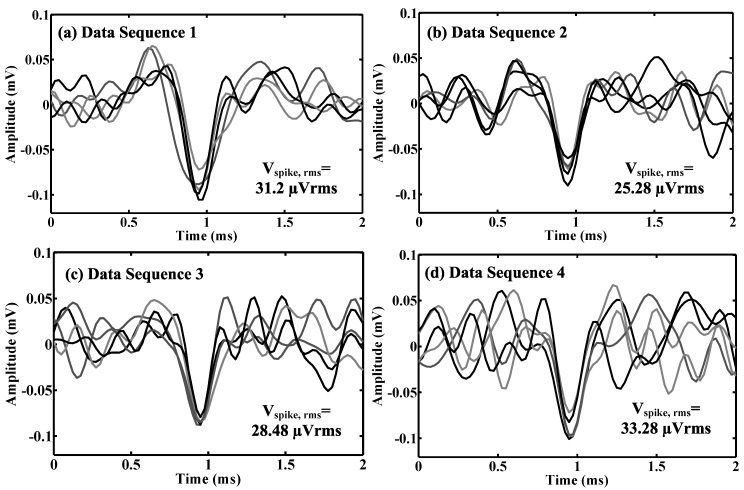
Exampled spike templates extracted from four *in vivo* data sequences (**a**–**d**).

The formula to estimate the data dynamic range is shown below:

(1) $$DR\left(dB\right)=20{\text{log}}_{10}\left|\frac{V_{peak-peak}}{V_{spike,rms}/16}\right|$$

where DR(dB) is dynamic range in dB, $V_{peak-peak}$ is the data amplitude and $V_{spike,rms}$ is the root mean-square (RMS) amplitude of a labeled spike template. The effective number of bits (*ENOB*) of dynamic range DR(bit) can be calculated as DR(bit)=[DR(dB)−1.76]/6.02. A few example spike templates extracted from four *in vivo* data sequences are shown in Figure 3. We assume that the spike template is digitalized at 16 levels, thus having sufficient resolution for neural signal processing. As shown in Table 1, the estimated data dynamic range based on the recordings from *in vivo* preparations and epilepsy patients are (12.06–13.58)-bit and (11.73–13.09)-bit, respectively.

**Table 1:** Averaged data dynamic range of *in vivo* neural activities.

Parameter	*In Vivo* Neural Data (1 Hz–10 kHz)	Epilepsy Patient Data (0.5 Hz–9 kHz)
Dynamic range without artifacts	(10.57 ± 0.32)-bit	(9.55 ± 0.31)-bit
Dynamic range with artifacts	(12.82 ± 0.76)-bit	(12.41 ± 0.68)-bit
Increase in dynamic range	2.25-bit	2.86-bit

## 3. ADC Architecture Comparison and Selection

**Figure 4:**
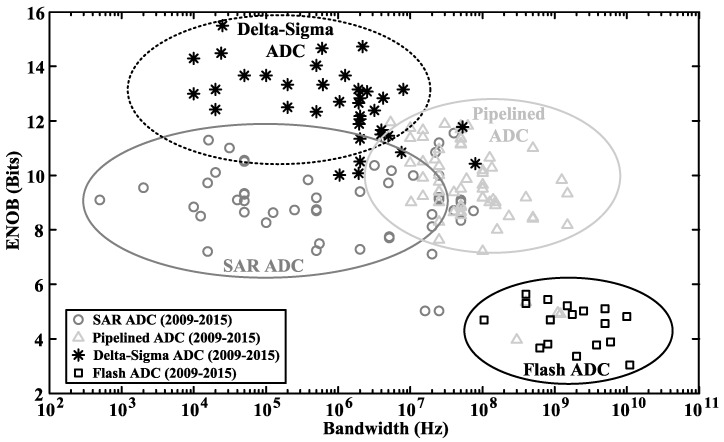
Performance comparison of ADC works published in the literature [33].

**Figure 5:**
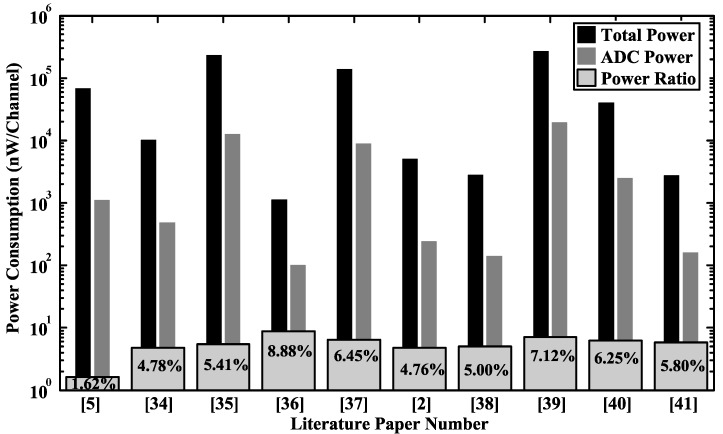
The total power consumption of the ADC block and its power ratio [2,5,34,35,36,37,38,39,40,41]. The power ratio is referred to as the ADC power divided by the total sensor power.

Figure 4 shows a survey of ADC designs published in the literature [33], including successive approximation register (SAR) ADC, flash ADC, pipelined ADC and Delta-Sigma ADC. Among the surveyed designs, the power consumption and power ratio (ADC power divided by the total power consumption) of neurosensors are shown in Figure 5 [2,5,34,35,36,37,38,39,40,41], where the maximum power ratio is less than 10%, and the total power consumption includes the power of instrumentation amplifier (IA), ADC, digital signal processor, power management circuits, and so on. The low power ratio suggests that there is some power budget trade-off for precision. Among the surveyed ADCs, flash and pipelined ADCs tend to have better performance in high speed (>20 MS/s) and medium resolution ((4–10)-bit) applications. The SAR ADCs typically provide an (8–10)-bit resolution [2,5,34,35,36,37,38], which is not enough to simultaneously record LFPs, extracellular spikes and artifacts without causing system saturation. Delta-Sigma ADCs can provide higher precision, thus avoiding saturation by using small gain in the IA. However, the costs are extra circuit power and area. In Section 4 and Section 5, we will focus on circuit design techniques for optimizing Delta-Sigma ADC to make it suitable for neural recordings.

## 4. Multi-Bit Switched Op-Amp-Based Delta-Sigma Modulator Design

### 4.1. Delta-Sigma Modulator Topology

**Figure 6:**
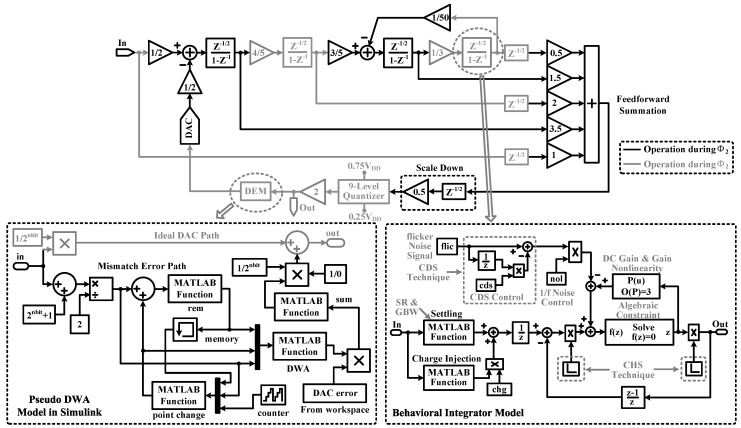
Block diagram of the proposed Delta-Sigma modulator with the behavioral integrator model [43,44] and the pseudo data weighted averaging (DWA) model.

The proposed Delta-Sigma modulator topology is shown in Figure 6, where a number of design choices are made for improving circuit performance. First, we have used a fully-feed-forward loop filter instead of a feedback one, because it gives extra signal paths from the outputs of integrators to the quantizer, so most of the signal energy is prevented from leaking into the modulator loop. Second, a multi-bit quantization technique and a local resonator with a coefficient of 1/50 are used for reducing in-band quantization noise, which in turn increases the ADC precision. Third, the switched-capacitor (SC) integrator is powered off during the sampling phase and powered back on during the integration phase. Hence, it can reduce the amplifier power consumption by half. Fourth, to suppress harmonic distortions caused by capacitor mismatches in the feedback digital-to-analog converter (DAC), a pseudo data weighted averaging (DWA) block is used to provide several dB boosting in dynamic range [42]. Fifth, the gain coefficient of the feed-forward summation block is scaled down by half to reduce its output swing, which can effectively allow one to adopt high-density MOS capacitors to simultaneously achieve a small circuit area and good linearity. Last, substantial processing time has been reserved for key blocks through clock scheduling. Due to these circuit design techniques, the proposed modulator topology is supposed to be suitable for ultra-low power high-precision applications [31].

### 4.2. Design Analysis on Non-Ideal Factors

This section gives the detailed analyses of the proposed modulator. A number of circuit design issues, including slew rate (*SR*), gain bandwidth (*GBW*), voltage gain, nonlinearity, charge injection, kT/C noise and 1/f noise, are presented for discussion.

#### 4.2.1. Finite Amplifier Gain

The noise transfer function (*NTF*) of the proposed modulator can be calculated as:

(2) $$NTF=\frac{{\left(z-1\right)}^{2}\left[{\left(z-1\right)}^{2}+0.0067\right]}{\left[\left(z+0.75\right)\left(z-1\right)+0.8\right]\left[{\left(z-1\right)}^{2}+0.0067\right]+0.24z\left(1.5z-1.333\right)}$$

When considering the finite amplifier gain in each integrator, the *NTF* becomes:

(3) $$NTF=\frac{\left(z-p_{1}\right)\left(z-p_{2}\right)\left[\left(z-p_{3}\right)\left(z-p_{4}\right)+0.0067p_{3}p_{4}\right]}{\left[\left(z+0.75p_{1}\right)\left(z-p_{2}\right)+0.8p_{1}p_{2}\right]\left[\left(z-p_{3}\right)\left(z-p_{4}\right)+0.0067p_{3}p_{4}\right]+M}$$

where $M=0.24p_{1}p_{2}p_{3}z\left(1.5z-1.333p_{4}\right)$, $p_{i}=1-1/A_{i}$ and $A_{i}$ is the finite voltage gain of the *i*-th integrator (i=1−4).

**Figure 7:**
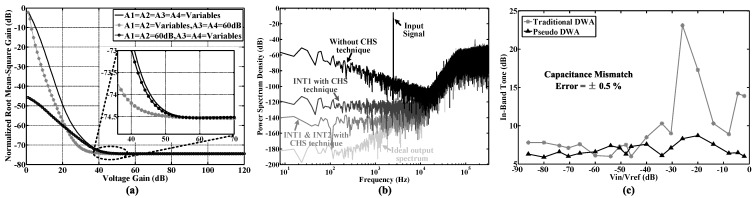
Simulation results of the proposed Delta-Sigma modulator. (**a**) The normalized RMS gain of the proposed noise transfer function (*NTF*) *versus* different voltage gains; (**b**) The modulator output spectrum with/without the chopper stabilization (CHS) technique in each integrator; (**c**) In-band tone performance with pseudo/traditional DWA. The capacitor mismatch error of the feedback digital-to-analog converter (DAC) is set to be ±0.5%.

Figure 7a shows the normalized RMS gains of the *NTF* with different amplifier gains. For the solid curve, the voltage gain of each operational transconductance amplifier (OTA) is set to be equal and scanned from 0 dB–120 dB. For the dotted line with “*”, the voltage gains of the first two stage OTAs are scanned from 0 dB–120 dB, while the gains of the third and fourth stage OTAs are fixed at 60 dB. For the dotted curve with “∘”, the voltage gains of the third- and fourth-stage OTAs are scanned from 0 dB–120 dB, while the gains in the first two stages are fixed at 60 dB. According to the simulation results, the OTA gains in the first two stages can be reduced to 40 dB, while the OTA gains in the third and fourth stages should be more than 60 dB.

#### 4.2.2. Chopper Stabilization

As shown in Figure 7b, low frequency noise can compromise ADC precision if not removed, especially for 1/f noise. Among different methods to remove low frequency noise, chopper stabilization (CHS) and correlated double sampling (CDS) techniques are popularly used [45,46,47]. In this design, we choose CHS, because CDS requires the amplifier to be constantly powered on, thus consuming more power. Figure 6 gives the integrator model with parameters, including finite gain, gain nonlinearity, finite *SR*, finite *GBW*, 1/f noise, switch-on resistance, charge injection, CDS, CHS, *etc*. Figure 7b shows the power spectrum at the modulator output. Through simulation, it is found that CHS is required in the first stage for achieving a 14-bit conversion precision, while this technique will be required for every stage when the target is to achieve a higher precision.

#### 4.2.3. Pseudo Data Weighted Averaging

A pseudo DWA behavioral block as shown in Figure 6 is added to analyze the effect of capacitor mismatch. The model can be explained as follows.

(4) $$V_{DAC}=V_{ideal,DAC}+e_{cap}$$

where $V_{DAC}$ and $V_{ideal,DAC}$ are the actual and ideal output of the feedback DAC, respectively; $e_{cap}$ is capacitor mismatch error, which has been processed by the pseudo DWA algorithm.

Figure Figure 7c gives the in-band tone performance with a capacitor mismatch error of ±0.5% in the feedback DAC. Compared to the traditional DWA, pseudo DWA can effectively suppress in-band harmonic distortions (<8 dB) to optimize the *SNDR* performance over the required bandwidth.

## 5. Circuit Design and Implementation

### 5.1. High Density MOS Capacitors

**Figure 8:**
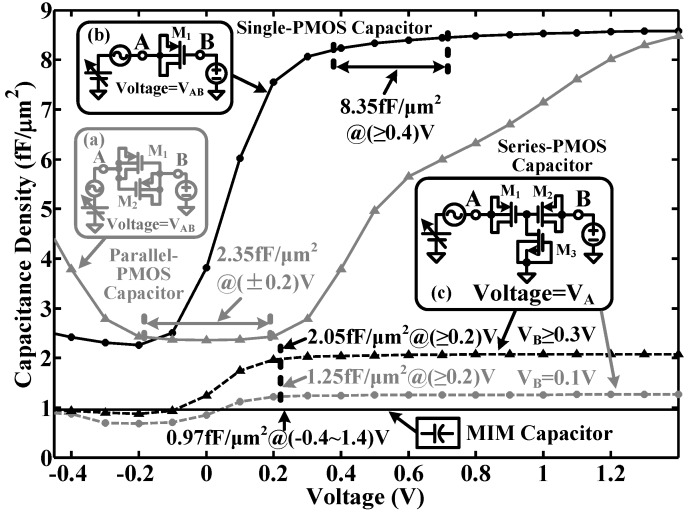
Simulated capacitance-voltage (C-V) curves of the proposed MOS capacitors in a 0.18-μm CMOS process: (**a**) parallel-PMOS type in the depletion region; (**b**) single-PMOS type and (**c**) series-PMOS type in the accumulation region.

Considering efficiency in terms of area, MOS capacitors are preferred due to their higher capacitance density [30]. Figure 8 illustrates three MOS capacitor circuits: (a) the parallel p-channel MOS (PMOS) type in the depletion region; (b) the single-PMOS type in the accumulation region and (c) the series-PMOS type in the accumulation region. The equivalent capacitances of the three MOS capacitors are shown as follows.

(5a) $$C_{eq-a}\approx C_{ox,M_{1}}\left|\right|C_{Depl,M_{1}}+C_{ox,M_{2}}\left|\right|C_{Depl,M_{2}}$$

(5b) $$C_{eq-b}\approx C_{ox,M_{1}}$$

(5c) $$C_{eq-c}\approx C_{ox,M_{1}}\left|\right|C_{ox,M_{2}}$$

where $C_{eq}$ is equivalent capacitance and $C_{ox}$ and $C_{Depl}$ are the gate-oxide capacitance and depletion capacitance of a transistor, respectively.

For circuit analysis, $C_{eq}$ can be analytically approximated as:

(6) $$C_{eq}=\frac{\int_{0}^{\frac{T_{s}}{2}}I_{MCAP}dt}{\int_{0}^{\frac{T_{s}}{2}}V_{MCAP}dt}$$

where $T_{s}$, $I_{MCAP}$ and $V_{MCAP}$ are periodic time, instantaneous AC current and instantaneous AC voltage of the input sinusoid waveform, respectively.

Figure 8 shows the simulated capacitance-voltage (C-V) curves of the three MOS capacitors in a 0.18-μm complementary MOS (CMOS) process. The single-PMOS type is able to provide a high density (8.45 fF/μm^2^) at a moderate bias voltage range (0.4 V–1.4 V) and poor linearity (<4.1%). The parallel-PMOS type gives a moderate density (2.35 fF/μm^2^) at a narrow bias voltage range (±0.2 V) and moderate linearity (<3.5%). The series-PMOS type has a moderate density (2.05 fF/μm^2^) with a wide bias voltage range (0.2 V–1.4 V) and good linearity (<2.8%). The density of the MIM capacitor is only 0.97 fF/μm^2^, though it allows a wide bias voltage range (−0.4 V–1.4 V) and good linearity (<0.1%).

**Figure 9:**
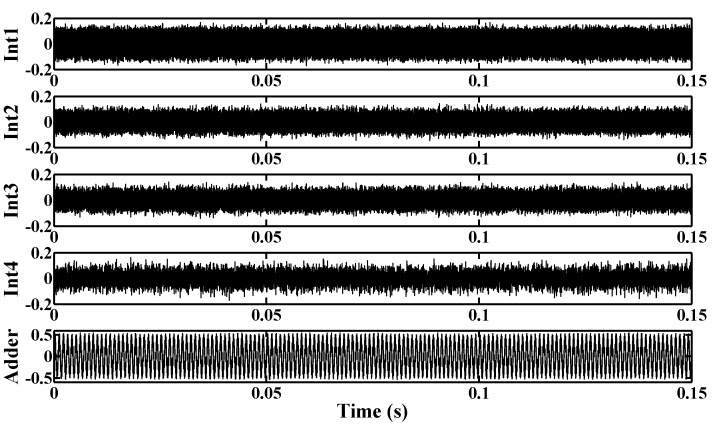
Simulated output swings of several circuit blocks in the proposed modulator.

Figure 9 shows the normalized output swings of several circuit blocks in the proposed modulator when the input is supplied with a −3.0 dB sinusoid input. It shows that the largest output swing in the feed-forward summation block is about 0.5 $V_{pp}$ (0.25 V–0.75 V), while the output of the integrators is less than 0.3 $V_{pp}$ (0.35 V–0.65 V). The simulation results suggest that the proposed MOS capacitor structures can be used without causing major distortions.

### 5.2. Fully-Clocked Power-Efficient Switched Op-Amp

**Figure 10:**
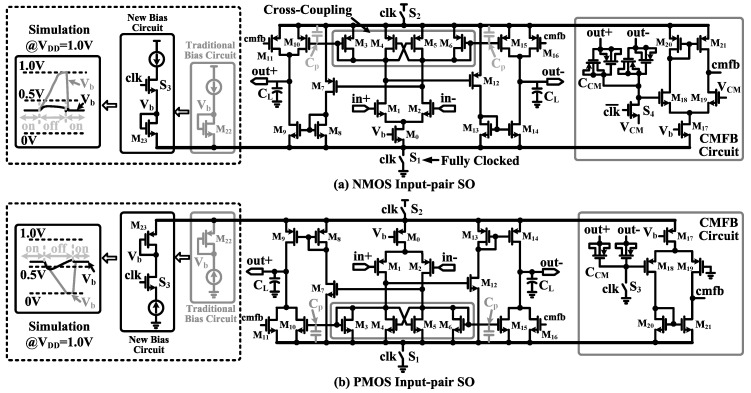
Fully-clocked power-efficient switched op-amps with the corresponding common mode feedback (CMFB) circuit: (**a**) NMOS input pair and (**b**) PMOS input pair.

Figure 10 shows the circuit schematics of the proposed fully-clocked current-mirror switched op-amps (NMOS input-pair type and PMOS input-pair type). It is a load-compensated single-stage OTA with a Class-AB output stage. A cross-coupling structure ($M_{3}$–$M_{6}$) is built in to achieve a large voltage gain and fast recovery from the off state. To save power, switches $S_{1}$ and $S_{2}$ are added to fully turn off the switched op-amp during the sampling phase. With a traditional bias circuit (highlighted in gray color, as shown in Figure 10), $V_{b}$ would be pulled to either $V_{DD}$ (NMOS input-pair switched op-amp) or ground (PMOS input-pair switched op-amp) during the off state. When the switched op-amp is turned on, there could be large instantaneous current and power consumption, which can be prevented by adding a series switch $S_{3}$ to reduce the voltage fluctuation of $V_{b}$ (<100 mV). With the proposed bias circuit, the power consumption of the switched op-amps can be reduced by 24% (2.4 μA).

To reduce circuit power and noise, it is important to select an appropriate switched op-amp for each integrator. The simulation results of both NMOS input-pair switched op-amp and PMOS input-pair switched op-amp are shown in Table 2, where the amplifier gain *A*, GBW, total current power consumption $I_{total}$ and amplifier input-referred noise ${\bar{V_{n}}}^{2}\left(f\right)$ are shown.

**Table 2:** Simulation results of the proposed switched op-amps.

Parameter	NMOS Input-Pair Switched Op-Amp	PMOS Input-Pair Switched Op-Amp
Gain *A*	61.1 dB	57.7 dB
Phase Margin	58.0^∘^	50.8^∘^
GBW	5.1 MHz	4.9 MHz
$C_{L}$	4 pF	4 pF
Current Power $I_{total}$	3.0 μA at 1.0 V	3.4 μA at 1.0 V
Noise Power	17.8 μV at (1–10 k) Hz	70.8 μV at (1–10 k) Hz
	5.0 μV at (150 k–160 k) Hz	15.8 μV at (150 k–160 k) Hz
*FOM*	6800 MHz·pF/mA	5765 MHz·pF/mA

The drain current ($I_{ds}$) of input-pair transistors ($M_{1}$ and $M_{2}$) is calculated as:

(7a) $$I_{ds,M_{1},n-type}=I=\frac{1}{2}{\text{μ}}_{n}C_{ox}\frac{W_{1}}{L_{1}}{\left(V_{gs1}-V_{thn}\right)}^{2}$$

(7b) $$I_{ds,M_{1},p-type}=I=\frac{1}{2}{\text{μ}}_{p}C_{ox}\frac{W_{1}}{L_{1}}{\left(V_{gs1}-V_{thp}\right)}^{2}$$

where *I* is the reference current, $V_{gs}$ is the voltage difference between the gate terminal and the source terminal and *W* and *L* are the transistor width and length; $V_{thn}$ and $V_{thp}$ are the transistor threshold voltage; ${\text{μ}}_{n}$ and ${\text{μ}}_{p}$ are the electron mobility and hole mobility, respectively. As ${\text{μ}}_{n}$ is larger than ${\text{μ}}_{p}$, $\left(V_{gs1}-V_{thn}\right)$ needs to be smaller than $\left(V_{gs1}-V_{thp}\right)$ to achieve the same drain current $I_{ds}$.

The voltage gain *A* and gain bandwidth GBW can be calculated as:

(8a) $$A_{n-type}\approx \frac{4}{\left(V_{gs1}-V_{thn}\right)\left({\text{λ}}_{n}+{\text{λ}}_{p}\right)}\frac{1}{\left(1-r\right)}$$

(8b) $$A_{p-type}\approx \frac{4}{\left(V_{gs1}-V_{thp}\right)\left({\text{λ}}_{n}+{\text{λ}}_{p}\right)}\frac{1}{\left(1-r\right)}$$

(9a) $$GBW_{n-type}\approx \frac{4BI}{\left(V_{gs1}-V_{thn}\right)C_{L}}$$

(9b) $$GBW_{p-type}\approx \frac{4BI}{\left(V_{gs1}-V_{thp}\right)C_{L}}$$

where $C_{L}$ is load capacitance, *B* is the current mirror ratio, *r* is the current starving ratio in the cross-coupling structure and ${\text{λ}}_{n}$ and ${\text{λ}}_{p}$ are the channel length modulation coefficients.

Given $\left(V_{gs1}-V_{thn}\right)$ < $\left(V_{gs1}-V_{thp}\right)$, it is found from Equations (8) and (9) that both $A_{n-type}$ and $GBW_{n-type}$ are larger than $A_{p-type}$ and $GBW_{p-type}$, respectively.

The input-referred noise ${\bar{V_{n}}}^{2}\left(f\right)$ of the proposed switched op-amps, including 1/f noise and thermal noise, can be approximately written as:

(10a) $${\bar{V_{n,n-type}}}^{2}\left(f\right)\approx \frac{2K}{C_{ox}W_{1}L_{1}f}\left[1+\frac{{\text{μ}}_{p}}{{\text{μ}}_{n}}{\left(\frac{L_{1}}{L_{3}}\right)}^{2}\right]+\frac{16kT}{3\sqrt{2{\text{μ}}_{n}C_{ox}\frac{W_{1}}{L_{1}}I}}\left[1+\frac{\left(\sqrt{r}+\sqrt{1-r}\right)}{\sqrt{\frac{{\text{μ}}_{n}}{{\text{μ}}_{p}}\frac{W_{1}L_{3}}{W_{3}L_{1}}}}\right]$$

(10b) $${\bar{V_{n,p-type}}}^{2}\left(f\right)\approx \frac{2K}{C_{ox}W_{1}L_{1}f}\left[1+\frac{{\text{μ}}_{n}}{{\text{μ}}_{p}}{\left(\frac{L_{1}}{L_{3}}\right)}^{2}\right]+\frac{16kT}{3\sqrt{2{\text{μ}}_{p}C_{ox}\frac{W_{1}}{L_{1}}I}}\left[1+\frac{\left(\sqrt{r}+\sqrt{1-r}\right)}{\sqrt{\frac{{\text{μ}}_{p}}{{\text{μ}}_{n}}\frac{W_{1}L_{3}}{W_{3}L_{1}}}}\right]$$

where *K* is a process-dependent constant on the order of $10^{-25}\;V^{2}F$; *k* is a Boltzmann constant with a value of 1.38 × $10^{-23}\;J\cdot K^{-1}$. Given the same ratio of $\frac{W_{i}}{L_{i}}$ (i=0−14), Equation (10) suggests that the NMOS input-pair switched op-amp has lower input-referred noise.

The total current power consumption of the proposed switched op-amps is calculated as:

(11a) $$I_{total,n-type}=2\left[1+\left(1+B\right)\left(1-r\right)\right]I+I_{cmfb}+I_{b}$$

(11b) $$I_{total,p-type}=2\left[1+\left(1+B\right)\left(1-r\right)\right]I+I_{cmfb}+I_{b}$$

where $I_{cmfb}$ is source current in the common mode feedback (CMFB) circuit and $I_{b}$ is bias current.

The *FOM* of an amplifier based on GBW, load capacitance $C_{L}$ and total current power consumption $I_{total}$ is shown below.

(12) $$FOM_{amp}=\frac{GBW*C_{L}}{I_{total}}$$

The typical *FOM* of a power-efficient OTA [28,30] is 4000–4500 MHz·pF/mA. As a comparison, the *FOM* of the proposed switched op-amp reaches 6800 MHz·pF/mA, corresponding to 50% increment.

### 5.3. Static Power-Less Area-Efficient Quantizer

**Figure 11:**
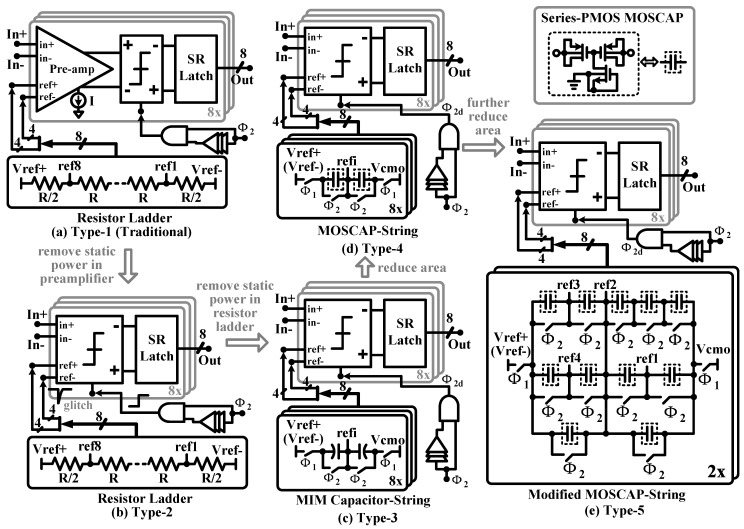
Circuit schematics of five nine-level quantizer structures. (**a**) Type 1: one resistor ladder and eight comparators with pre-amps; (**b**) Type 2: one resistor ladder and eight comparators without pre-amps; (**c**) Type 3: eight metal-insulator-metal (MIM) capacitor strings and eight comparators without pre-amps. (**d**) Type 4: eight MOS capacitor strings and eight comparators without pre-amps. (**e**) Type 5: two MOS capacitor strings and eight comparators without pre-amps.

As shown in Figure 11a, a flash ADC consisting of a resistor ladder and comparators with pre-amps is a popular choice for implementing a quantizer, but its drawbacks are large circuit power and area caused by the resistor ladder and pre-amps. A dynamic comparator without pre-amps, as shown in Figure 11b, can be used to remove the static power. However, due to the effect of clock kick-back, large glitches appear at the reference-input terminals, which may give incorrect comparison results and degrade system performance. Instead of a resistor ladder, MIM capacitor strings in Figure 11c can be a remedy to eliminate the glitches and generate the required voltage references. In this case, the static power consumed by both the comparators and resistor ladder is removed. Two non-overlapping clock signals (${\text{Φ}}_{1}$ and ${\text{Φ}}_{2}$) with their corresponding delayed versions (${\text{Φ}}_{1d}$ and ${\text{Φ}}_{2d}$) are used to stabilize the voltage references generated by the MIM capacitor strings before the comparators give the comparison results. To further reduce the circuit area, the MIM capacitor is replaced by the high density series-PMOS structure, as shown in Figure 11d. After optimizing the reference circuit, the number of unit capacitors in the MOS capacitor strings is reduced, as shown in Figure 11e.

**Figure 12:**
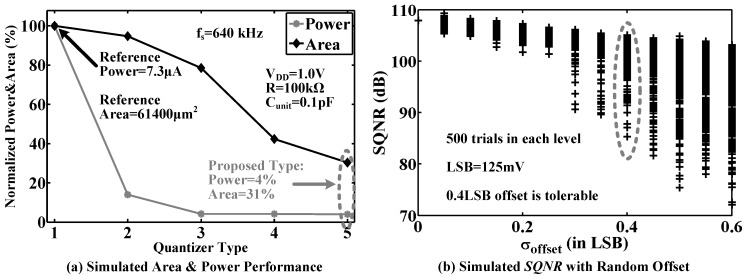
(**a**) Simulated area and power performance of the introduced five nine-level quantizers; (**b**) Simulated signal-to-quantization noise ratio (*SQNR*) with random comparator offset, where there are 500 trials at each level.

Figure 12a shows the simulated power and circuit area of the quantizers in Figure 11a–e. Compared to the traditional structure in Figure 11a, the proposed quantizer saves power consumption by 96% and reduces circuit area by 69%. Figure 12b gives the effect of random comparator offset. To achieve a 85-dB signal-to-quantization noise ratio (*SQNR*), the random offset with a standard deviation of up to 0.4 LSB (equal to 50 mV) can be easily accommodated in the proposed modulator.

### 5.4. Novel Power- and Area-Efficient Resonator

**Figure 13:**
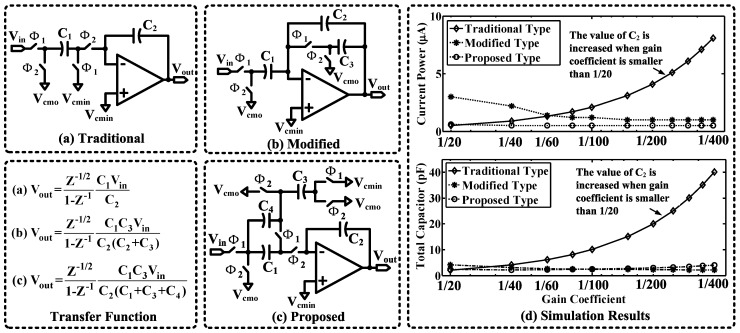
The circuit schematics and simulation results of three resonator structures ((**a**) traditional, (**b**) modified and (**c**) proposed). (**d**) Simulated power and area performance of the resonators. For a fair comparison, the integration capacitance $C_{2}$ and the unit capacitance are assumed to be 2 pF and 0.1 pF, respectively. Furthermore, the slew rate (*SR*) of the operational transconductance amplifier (OTA) is assumed to be 2 V/μs.

A local resonator scheme with a coefficient of 1/50 is applied to reduce in-band quantization noise power. Figure 13a–c show the resonator schematics, and their corresponding transfer functions are listed below.

(13a) $$H{\left(z\right)}_{traditional-type}=\frac{C_{1}}{C_{2}}\frac{z^{-1/2}}{1-z^{-1}}$$

(13b) $$H{\left(z\right)}_{modified-type}=\frac{C_{1}C_{3}}{C_{2}\left(C_{2}+C_{3}\right)}\frac{z^{-1/2}}{1-z^{-1}}$$

(13c) $$H{\left(z\right)}_{proposed-type}=\frac{C_{1}C_{3}}{C_{2}\left(C_{1}+C_{3}+C_{4}\right)}\frac{z^{-1/2}}{1-z^{-1}}$$

The traditional resonator as shown in Figure 13a can be used to realize a coefficient larger than 1/20, where the amplifier can be turned off during ${\text{Φ}}_{1}$. To implement a smaller coefficient, the traditional resonator will take severe penalties in power and area. Figure 13b shows a modified resonator that was reported in [48] for implementing a notch filter. This modified resonator can achieve small coefficients without requiring much extra circuit area. However, it is not applicable to the switched op-amp technique, because the amplifier has to be constantly turned on. As a comparison, we propose a novel design as shown in Figure 13c, which can implement a wide range of coefficients, yet achieve good efficiency in terms of power and area.

### 5.5. Complete Modulator Circuit

**Figure 14:**
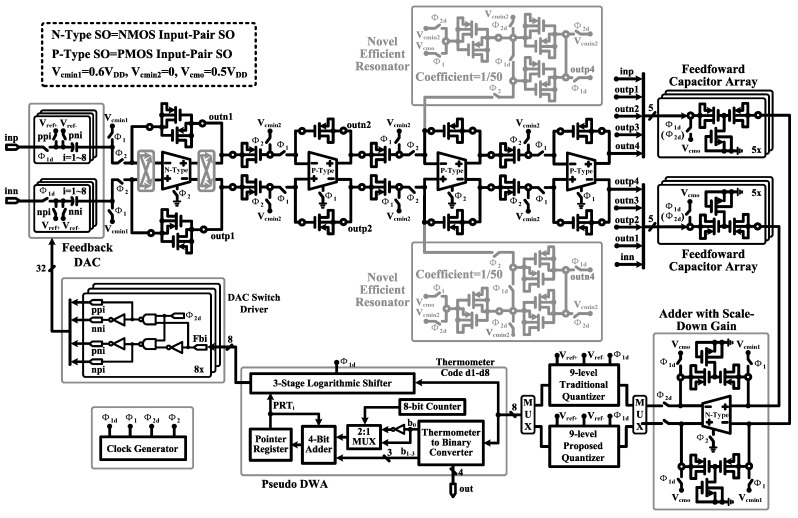
Circuit schematic of the proposed modulator. The amplifiers in the first-stage integrator and feed-forward signal summation block are designed with NMOS input-pair switched op-amp, while other amplifiers are designed with PMOS input-pair switched op-amp. A DAC switch driver is adopted to reduce the number of the feedback signalsfrom pseudo DWA.

Figure 14 shows the circuit implementation of the proposed fourth-order feed-forward Delta-Sigma modulator. We have done a careful selection of appropriate switched op-amp structure and MOS capacitor type for each integrator. (1) Because the input-referred noise of the first-stage integrator is directly transferred to the modulator output with a closed-loop gain of near 1 V/V, the NMOS input-pair switched op-amp (low noise), the MIM capacitor (perfect linearity) and the parallel-PMOS capacitor (moderate density) are used. These allow a lower noise floor, a smaller distortion and a higher area efficiency. (2) The NMOS input-pair switched op-amp and series-PMOS capacitor (wide bias voltage range) are adopted in the feed-forward signal summation block to meet the requirement of near full-scale input range inp or inn. In order to suppress the harmonic distortions caused by the series-PMOS capacitor, the output signal swing of this block is scaled down by 50%. (3) For other integrators, a single-PMOS capacitor (high density) is employed to improve the area efficiency effectively. To make sure that the single-PMOS capacitor operates in the saturation region and has good linearity, PMOS input-pair switched op-amp is used, and the input common mode voltage $V_{cmin2}$ is set to be 0 V. (4) To improve the *SQNR*, a novel area- and power-efficient resonator scheme using a parallel-PMOS capacitor is proposed to realize a small coefficient of 1/50. Compared to a modulator using only an MIM capacitor, 55% capacitor area is saved in the proposed modulator design.

## 6. Measurement Results

**Figure 15:**
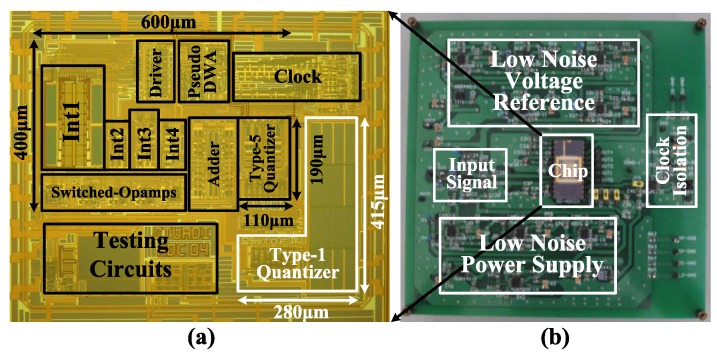
(**a**) Chip photo of the proposed Delta-Sigma modulator; (**b**) Circuit testing board.

**Figure 16:**
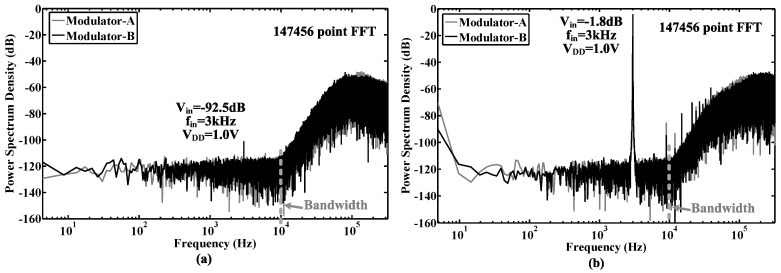
Measured output spectrums of the modulators *versus* different input amplitudes. (**a**) The input is a −92.5-dB, 3-kHz sinusoidal waveform with respect to a 1.0-V reference; (**b**) The input is a −1.8-dB, 3-kHz sinusoidal waveform with respect to a 1.0-V reference.

**Figure 17:**
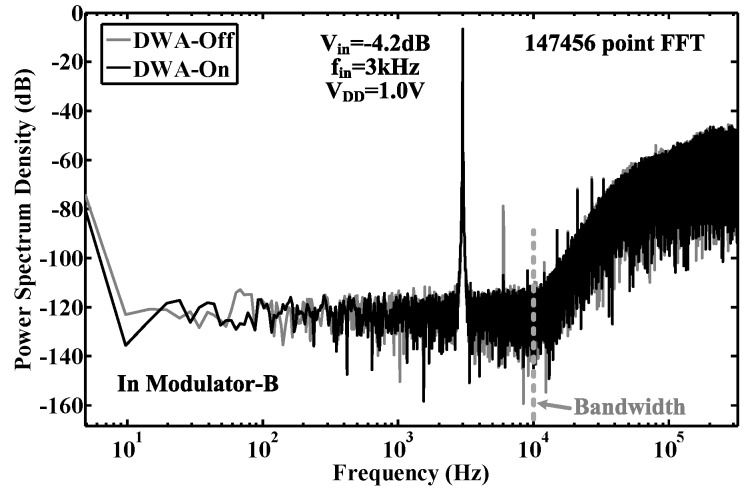
Measured output spectrum of Modulator B with/without pseudo DWA. The input is a −4.2-dB, 3-kHz sinusoidal waveform with respect to a 1.0-V reference.

**Figure 18:**
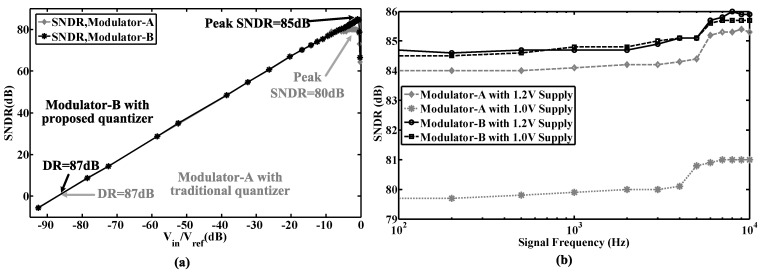
(**a**) Measured *SNDR* at different input amplitudes; (**b**) Measured peak *SNDR* at different frequencies and supply voltages.

The proposed modulator was fabricated in a 0.18-μm CMOS process. The chip micrograph and circuit testing setup are shown in Figure 15, where the core area is 0.4 mm × 0.6 mm. The traditional quantizer and proposed quantizer occupy an area of 280 μm × 415 μm and 110 μm × 190 μm, respectively. The input is provided by an Audio Precision signal generator, and the output is captured by a logical analyzer. There are two designs on this chip: Modulator A with the traditional quantizer and Modulator B with the proposed quantizer. Figure 16, Figure 17 and Figure 18 show the measurement results with a 1.0-V supply and a 640-kHz clock. Figure 16 demonstrates that the proposed modulator has an 87 dB dynamic range (14-bit) for digitizing biomedical signals and artifacts. Figure 17 shows that the pseudo DWA technique can noticeably suppress in-band harmonic distortions. Figure 18a gives the measured *SNDR* *versus* different input amplitudes. For Modulator A, the measured peak *SNDR* and dynamic range are 80 dB and 87 dB, respectively, while the total power consumption is 20 μW with an *FOM* of 122 fJ/conversion step. For Modulator B, the measured peak *SNDR* and dynamic range are 85 dB and 87 dB, respectively, while the total power consumption is 13 μW. These specifications correspond to an *FOM* of 45 fJ/conversion step. Figure 18b shows the measured peak *SNDR* at different signal frequencies and power supplies. Table 3 gives a performance summary in comparisonto other publications.

**Table 3:** Performance summary and comparison.

Modulator	[49]	[50]	[26]	[30]	[51]	[28]	[25]	This Work
Process (nm)	130	65	180	180	180	180	180	180
Integrator Type	Inverter	Comp.	SO	OTA	OTA	OTA	Inverter	SO
$V_{DD}$ (V)	1.2	1.2	0.7	0.7	1.8	1.0	0.7	1.0
$f_{s}$ (MHz)	1.28	40	5	1.024	6.144	4	4	0.64
Bandwidth (kHz)	20	2500	25	8	24	20	20	10
Power (μW)	165	3730	870	80	110	140	36	13
Dynamic Range (dB)	83.0	71.3	100	75	92.5	88	85	87
*SNDR* (dB)	72.5	70.4	95	67	88	81	81	85
*FOM* (fJ/conversion step)	1197	276	378.5	2733	111.6	381.7	98.1	45

Comp., comparator; SO, switched op-amp; OTA, operational transconductance amplifier.

## 7. Conclusions

Data analysis on recorded sequences from both *in vivo* preparations and epileptic patients suggests that a wide system dynamic range is required to simultaneously record both neural activities and artifacts. To meet the required dynamic range, yet low power operation, this paper presents design analyses, circuit implementation and measurement of a Delta-Sigma modulator chip. Powered by a 1.0-V supply, the chip can achieve an 85-dB peak *SNDR* and an 87-dB dynamic range when integrated over a 10-kHz bandwidth. The total power consumption of the modulator is 13 μW, which corresponds to an *FOM* of 45 fJ/conversion step. The competitive circuit specifications make this design a good candidate for building high precision neurosensors.
